# Case Report: Zellweger Syndrome and Humoral Immunodeficiency: The Relevance of Newborn Screening for Primary Immunodeficiency

**DOI:** 10.3389/fped.2022.852943

**Published:** 2022-03-25

**Authors:** C. Fazi, L. Lodi, L. Magi, C. Canessa, M. Giovannini, C. Pelosi, F. Pochiero, E. Procopio, M. A. Donati, C. Azzari, S. Ricci

**Affiliations:** ^1^Pediatric Immunology Division, Meyer Children's Hospital, Florence, Italy; ^2^Department of Health Sciences, University of Florence, Florence, Italy; ^3^Neonatology Division, San Donato Hospital, Arezzo, Italy; ^4^Pediatric Allergy Division, Meyer Children's Hospital, Florence, Italy; ^5^Department of Metabolic Diseases, Meyer Children's Hospital, Florence, Italy

**Keywords:** Zellweger, humoral immunodeficiency, metabolic disease, B cell deficiency, newborn screening (NBS), inborn error of immunity (IEI), primary immunodeficiency

## Abstract

**Background:**

Zellweger syndrome (ZS) is a congenital autosomal recessive disease within the spectrum of peroxisome biogenesis disorders, characterized by the impairment of peroxisome assembly. The presence of peroxisome enzyme deficiencies leads to complex developmental sequelae, progressive disabilities, and multiorgan damage, due to intracellular accumulation of very-long-chain fatty acids (VLCFAs).

**Case Presentation:**

We report the case of an infant affected by ZS in which agammaglobulinemia, detected through neonatal screening of congenital immunodeficiencies, appeared as a peculiar trait standing out among all the other classical characteristics of the syndrome. The exome analysis through next-generation sequencing (NGS), which had previously confirmed the diagnostic suspicion of ZS, was repeated, but no mutations causative of inborn error of immunity (humoral defect) were detected.

**Conclusion:**

In this case, no genetic variants accountable for the abovementioned agammaglobulinemia were detected. Given that the scientific literature reports the involvement of peroxisomes in the activation of Nuclear Factor κ-light-chain-enhancer of activated B cells (NF-κB) pathway, which is crucial for B-cell survival, with this work, we hypothesize the existence of a link between ZS and humoral immunodeficiencies. Further studies are required to confirm this hypothesis.

## Introduction

### Zellweger Syndrome

Zellweger syndrome (ZS) is a severe neurodegenerative disease that belongs to the spectrum of peroxisome biogenesis disorders (PBDs) (1).

Peroxisomes are membrane-bound organelles that play a vital role in a broad spectrum of cellular metabolic pathways. Their assembly requires a set of proteins termed “peroxins,” and the presence of biallelic pathogenic mutations in any one of 14 genes coding for peroxins (“PEX” genes) negatively affects the correct peroxisome assemblage (2), leading to the formation of functionally incompetent peroxisomes. This causes the intracellular accumulation of very-long-chain fatty acids (VLCFAs) and the damage of most of the developing organs such as the liver, bone, kidneys, and the organizing brain (1, 3).

ZS is commonly caused by mutations in PEX1 and PEX6 genes (1), and it is characterized by distinctive craniofacial dysmorphisms, multiorgan damage, and neuronal migration defects (4), which are due to the abnormal levels of VLCFAs accumulated in the body.

Clinical suspicion of PBDs requires confirmation through the measurement of plasma VLCFA levels, which tend to rise in the blood, urine, and cells: the elevation of C26:0 and C26:1 fatty acids and the higher ratio of C24:0/C22:0 and C26:0/C22:0 are, in fact, consistent with peroxisomal fatty acid β-oxidation defect (4). Abnormal values in VLCFAs mandate further testing, including repetition of VLCFA measurement on a plasma sample after overnight fasting and analysis of other peroxisomal markers (phytanic acid, pristanic acid, plasmalogens, docosahexaenoic, primary bile acids, etc.) (1).

Genetic investigations intended to identify the specific gene mutations through a multi-gene panel, exome, or genome sequencing will definitively confirm or rule out the initial hypothesis. Moreover, genetic testing for family planning purposes should be considered (5). The average life expectancy is 1 year (6) since patients with ZS typically die at an early age due to prolonged apnea episodes, respiratory failure, or infectious complications (1).

For what concerns the interplay between peroxisomes and the immune system, Di Cara and colleagues (7) recently reviewed and summarized the last 10 years of discoveries on peroxisomes as metabolic mediators of immunity, but the association between peroxisomes and humoral defects has never been described so far.

In the present paper, we report for the first time the case of a pediatric patient with a confirmed diagnosis of ZS presenting a severe immunological humoral defect that could not be ascribed to other genetic mutations apart from the one that led to the diagnosis of the syndrome.

### B-Cell Deficiencies and Newborn Screening

B-cell immunodeficiencies are the most common type of immunodeficiencies, accounting for approximately 50% of all primary immunodeficiency (PID) diagnoses. They are characterized by either insufficient number of B cells or impaired functioning/differentiation of them, as well as the absence of serum immunoglobulins and/or loss of antibody function (8). They may be due to intrinsic B-cell defects, failure of interaction between B cells and T cells, loss of appropriate bone marrow responses, or defects of immune regulation (9).

The clinical presentation generally occurs after 6 months of age, and patients typically complain of recurrent and severe sino-pulmonary infections (otitis media, sinusitis, pneumonia, etc.), diarrhea, fatigue, and autoimmune manifestations such as autoimmune cytopenias (9).

B-cell immunodeficiencies are categorized into the following:

1) severe reduction in all serum immunoglobulin isotypes with profoundly decreased or absent B cells, recognized as agammaglobulinemia (e.g., X-linked agammaglobulinemia Bruton's agammaglobulinemia)
2) severe reduction in at least 2 serum immunoglobulin isotypes (typically IgG and IgA) with a normal or low number of B cells (common variable immunodeficiency (CVID) phenotype)
3) severe reduction in serum IgG and IgA with normal/elevated IgM with normal numbers of B cells (hyper IgM syndrome)
4) isotype or light chain deficiencies with generally normal numbers of B cells (9).

For what concerns the newborn screening (NBS), there is growing evidence that T-cell receptor excision circles (TRECs) and κ-deleting recombination excision circles (KRECs) quantification can be very useful in the early identification, protection, and treatment of patients with primary and acquired immune deficiencies (10). TRECs and KRECs are episomal DNA fragments resulting from gene rearrangements that occur during T- and B-lymphocyte maturation. Representing DNA biomarkers of the natural development of T and B lymphocytes, TRECs and KRECs have recently respectively been used for detection of T- or B-cell lymphopenia in neonates since they can be easily extracted from dried blood spots (DBS) on Guthrie cards and measured by real-time quantitative PCR (10).

KREC assay was initially developed to measure B-cell proliferation and its contribution to B-cell homeostasis (11), but it has recently been proposed as a bone marrow output marker (12, 13). In 2011, the utility of the KREC assay in identifying XLA and XLA-like diseases in neonates was demonstrated (14); however, there are instances of normal KRECs levels happening in some PIDs (15). Combined TREC/KREC assay has some advantages, as it allows identification of individuals with different forms of PID, which might be missed by TREC assay, including late-onset ADA, X-linked, or autosomal recessive agammaglobulinemia, and some cases of Nijmegen breakage syndrome (8).

## Case Presentation

We report the case of a newborn affected by ZS who was referred to the immunology unit of the Meyer Children's Hospital due to the positive result at the neonatal screening (NBS) for PID.

The patient of this case was a female infant born through vaginal spontaneous delivery at 39 + 1 weeks of gestational age from parents who, although not consanguineous, come from a small Macedonian village of 2,000 inhabitants. Her birth weight was 3,130 g, height 51 cm, and head circumference 33 cm. She was the third out of three pregnancies, and her elder siblings are in good health conditions. Deepening the infant's family history, it has been reported that the mother had previously undergone splenectomy due to immune thrombocytopenic purpura and that unexplained deaths had occurred in newborns from both the father's and the mother's side of the family.

Pregnancy had been progressing without any complication until the second trimester when routine ultrasound and, subsequently, magnetic resonance detected callosal hypoplasia as well as the presence of cavum vergae and bilateral ventriculomegaly.

At the first physical examination after birth, the baby presented with clear syndromic features, with craniofacial dysmorphisms, axial and appendicular severe hypotonia, bilateral horizontal eye nystagmus, and trigonocephaly as the most evident characteristics, but also arthrogryposis of both hands, single transverse palmar crease, and bilateral reducible talipes equinovarus were reported. Additionally, spontaneous movements were hardly detectable, tendon reflexes were absent, archaic reflexes could not be elicited, and, because of hypovalid suction, milk had to be delivered through gavage. Also, 10 days after birth, it proved necessary to start levetiracetam and phenobarbital therapy due to the occurrence of seizures.

Further examinations were therefore performed: cranial Magnetic Resonance Imaging (MRI). detected neocortical dysplasia with polymicrogyria of both frontal and parietal lobes, and abdominal sonography reported mild intrahepatic biliary ductal ectasia. Blood exams showed progressive hepatic failure (hepatic enzymes reached aspartate transaminase (AST) 783 U/L, alanine transaminase (ALT) 469 U/L, and gamma-glutamyltransferase (GGT) 222 U/L as maximal values before slowly decreasing) due to which vitamin K, ursodeoxycholic acid, and liposoluble vitamins were added to the basal therapy.

In light of the rising diagnostic suspicion of ZS, VLCFA examination was requested, and since the results were far over the normal range (C26 101 μmol/L, normal range 0.2–0.9 μmol/L; C24/C22 1.5 μmol/L, normal range 0.6–1.2 μmol/L; C26/C22 0.24 μmol/L, normal range 0–0.030 μmol/L), genetic testing with a panel of 14 genes associated with peroxisomal diseases was performed. A homozygous mutation (c.1314_1321delGGAGGCCT [p.(Glu439Glyfs^*^3)]) in PEX6 genes, inherited from both parents who are unaffected carriers, was identified, confirming the diagnosis.

The patient came to the attention of the Pediatric Immunology Unit at 5 days of age due to the positive result at NBS for PID: multiple RT-PCR for TRECs and KREC quantification on DBS clearly showed the complete absence of KRECs (normal value >10 copies/μl). The consequent study of lymphocyte subpopulations showed, in concordance with the previous findings, the complete absence of B lymphocyte (0.1%) and immunoglobulin G levels at the lower limit of the normal interval with the absence of IgA and low IgM (IgM 7 mg/dl, IgA <5 mg/dl, and IgG 277 mg/dl) (Table 1). In accordance with our patient's parents, replacement therapy with monthly subcutaneous immunoglobulin (IVIg) administration (400 mg/kg/dose) was initiated. Despite the initiation of immunoglobulin therapy at the age of 4 months, the IgG level was not significantly increased, probably testifying to a defect of output (Table 1).

**Table 1:** Patient's biochemical, hematological, and immunological data from birth until 3 months old.

	**Range**	**14 days old**	**1 month old**	**2 months old**	**4 months old**
RBC	3.1–4.5 × 10^6^/μl	3.71	3.93	3.75	4.16
HGB	10–14 g/dl	11.3	11.2	9.9	10.9
HCT	27%−42%	33.3	33.1	31.9	33.1
MCV	75–95 fl	89.8	84.2	85.1	79.6
MCH	26–35 pg	30.5	28.4	28.8	26.2
MCHC	28–36 g/dl	33.9	33.7	33	32.9
RDW	11.6%−16.5%	15.9	16.9	16.2	15.4
PLT	150,000–350,000/μl	Not available	135,000	213,000	533,000
MPV	7.1–10 fl	Not available	9.3	8.5	8.4
WBC	5,000–15,000 cell/μl	5,400	4,300	7,000	12,600
% Neutrophils	10%−50%	37.6	23	27.5	37.2
% Lymphocytes	40%−80%	49.3	67.7	56.1	43.3
CD3	2,500–5,500 cell/μl		3,279	4,016	4,944.9
% CD3	55%−80%		96	91	90.9
HLADR+	%		2.9		
CD4	1,600–4,000 cell/μl		2,879	3,073	3,884.1
%CD4	30%−50%		84	70	71.4
CD3+CD8+	190–1,140 cell/μl		343	874	
%CD3+CD8+	14%−38%		10	20	
CD45RO	%		16		
CD45RA+31+	%		69		
CD45RA+31+	%		82		
CD4/CD8	1.4–2.7		8.4	3.5	3.9
CD19	90–660 cell/μl		3	24	8
% CD19	6%−25%		0.1	0.5	0.14
CD56CD16+	5%−24%		110	329	446
% CD56CD16	8%−17%		3	8	8.2
IgG	232–1411 mg/dl	564	526	277	326 (after IRT)
IgM	5–145 mg/dl	0	1	7	5
IgA	5–83 mg/dl	1	<5	<5	<5
TREC on dried blood spot		Normally expressed	Normally expressed		
KREC on dried blood spot		No expression	No expression	No expression	
C26	0.2–0.9 μmol/L	8			
C24/C22	0.6–1.2 μmol/L	1.5			
C26/C22	0–0.030 μmol/L	0.24			
AST	8–60 UI/L	783		424	473
ALT	7–45 UI/L	469		162	190
Total bilirubin	0–1.2 mg/dl			0.24	0.28
Direct bilirubin	0–0.3 mg/dl			0.2	0.23
Gamma-GT	8–127 UI/L	222		143	130

*IRT, immunoglobulin replacement therapy; RBC, red blood cells; HGB, hemoglobin; HCT, hematocrit; MCV, mean corpuscular volume; MCH, mean corpuscular hemoglobin; MCHC, mean corpuscular hemoglobin concentration; RDW, red cell distribution width; PLT, platelet; MPV, mean platelet volume; WBC, white blood cell; TREC, T-cell receptor excision circle; KREC, κ-deleting recombination excision circle; AST, aspartate transaminase; ALT, alanine transaminase*.

Our patient died at the age of 5 months due to a cardiorespiratory arrest. Postmortem, in order to shed light on this complex clinical case and to dispel any doubt around the possibility of a genetic cause of agammaglobulinemia, the baby's exome was reanalyzed, but no known or unknown variants causative of inborn error of immunity were detected.

## Genetic Analysis

Written informed consent has been obtained from the parents of the patient for the publication of this case report.

In the search for the genetic cause of the syndromic features presented by the baby, quantitative fluorescence PCR (QF-PCR) was performed, and no aneuploidies in chromosomes 13, 18, 21, and X were identified.

Additionally, in order to confirm the suspicion of ZS, further genetic tests such as the whole-exome sequencing were requested, and a panel of 14 genes associated with peroxisomal diseases was analyzed: a homozygous mutation (c.1314_1321delGGAGGCCT [p.(Glu439Glyfs^*^3)]) in PEX6 genes was identified, confirming the diagnosis of ZS.

Postmortem, our patient exome was analyzed for a second time, but no mutations in the panel of genes responsible for hypo- or agammaglobulinemia were detected (BTK, IGHM, CD79A, CD79B, BLNK, IGLL1, TCF3, PIK3R1, PIK3CD, SLC39A7, TCF3, and TOP2B) (16). No significant *de novo* or homozygous variants were identified.

## Discussion

ZS is a severe neurodegenerative disease, which, along with neonatal adrenoleukodystrophy, infantile Refsum disease, and rhizomelic chondrodysplasia punctata, belongs to the spectrum of PBDs (1).

It is a syndrome inherited in an autosomal recessive manner; therefore, the presence of biallelic pathogenic mutations in any of 14 genes coding for peroxins [most commonly PEX 1, 66% of cases, and PEX6 genes (1)] leads to defects in peroxisomes assembly, causing the formation of functionally incompetent peroxisomes, which fail to perform their metabolic duties, including the beta-oxidation of fatty acids with a chain length of more than 22 carbons. The subsequent impairment of multiple catabolic and anabolic pathways results in the accumulation of VLCFAs, phytanic- and pristanic acid, C27-bile acid intermediates, and pipecolic acid in plasma and have a deficiency of plasmalogens in erythrocytes (17).

Clinically, ZS is characterized by distinctive craniofacial dysmorphisms, renal micronodular cortical cysts, which are usually asymptomatic (4), and enlarged liver with dysfunction of the hepatocellular and biliary systems. For what concerns neurological development, neuronal migration defects (4) determine a higher incidence of poorly controlled neonatal seizures and severe hypotonia, which is frequently associated with respiratory impairment and poor feeding. Brain MRI may detect polymicrogyria and heterotopias as well as brain atrophy, subependymal germinolytic cysts, medial pachygyria, and lateral polymicrogyria (18). Additionally, bone abnormalities such as chondrodysplasia punctata, eye diseases (cataracts, congenital glaucoma, retinal dystrophy, pigmentary retinopathy, optic atrophy, etc.), and sensorineural deafness can be present as well as cardiovascular malformations and pulmonary hypoplasia (3, 4, 19).

With regard to the connection between peroxisome dysfunction and impaired cellular development and survival, it is nowadays well known that peroxisomes produce factors that are essential to the survival and the correct functionality of numerous systems such as peripheral and central neurons, myocytes, hepatobiliary metabolism, and pancreatic islet beta cells. For what concerns the interplay between peroxisomes and the immune system, Di Cara and colleagues (7) recently reviewed and summarized the last 10 years of discoveries on peroxisomes as metabolic mediators of immunity: the literature revision, which consisted of two case reports, revealed a clinical association between ZS and immunodeficiency. The first report was written in 1974 by Gilchrist et al. (20), who described, in a letter published on *Lancet*, a defect in the differentiation and function of T cells in clinical cases of ZS. Afterward, in 2016, Cardoso et al. (21) described the case of a patient affected by ZS who drastically improved his state of profound T-lymphopenia after obtaining a full recovery of his nutritional status. Nevertheless, the association between peroxisomes and humoral defects has never been described so far.

According to many European and worldwide health policies, Tuscany is the only region in Italy that is performing to all the neonates (around 25,000 per year) the NBS for primary immunodeficiencies with TREC and KREC quantification on DBS through multiplex real-time PCR. This screening campaign started in October 2018 and the one described in this report is the second case in which a severe humoral defect was identified in the context of a very rare multisystem syndrome: the first one was an infant with combined immunodeficiency due to neuroblastoma-amplified sequence (NBAS) deficiency (22). What is more, the fact that the humoral defect was present at birth, having been detected by the abovementioned screening, and that the B-lymphocyte count did not rise during the follow-up, defines the immunological defect detected as primary and persistent (Table 1).

This is therefore the first report of a pediatric patient with a confirmed diagnosis of ZS presenting a severe immunological humoral defect, which could not be ascribed to any other known genetic mutation linked to immunological impairment, apart from the one leading to the diagnosis of ZS and any eventual intronic mutation, which could not be detected through the exome sequencing.

Interestingly, peroxisomes are described in scientific literature as producers of bioactive metabolites capable of driving immune signaling (23). Specifically, peroxisomes seem to have a role in the activation of the NF-κB pathway, which is known to be crucial for B-cell survival. In fact, the B-cell activation factor receptor belonging to the tumor necrosis factor superfamily (BAFF) provides critical survival signals to all splenic B-cell subsets, activating both canonical and non-canonical NF-κB pathways (24). We can therefore speculate that the severe defect of peroxisome function that characterizes the ZS can affect the NF-κB pathway activation and can, consequently, negatively impact the survival of B cells.

Even if every day more detailed International Union of Immunological Societies (IUIS) phenotypic classification (16) has not provided us with a clear association between ZS and immunological defects yet, we hold the view that the link between primary immunodeficiencies and metabolic disorders should not be undervalued. As a matter of fact, many other metabolic disorders are known to be the cause of immunological defects: adenosine-deaminase deficiency is, for instance, responsible for severe combined immunodeficiencies, familial hemophagocytic lymphohistiocytosis (e.g., Griscelli syndrome) is caused by alterations in the molecular mechanisms involved in vesicle transport and membrane trafficking processes, etc.

In light of all the above, as long as the characterization of peroxisome diseases is not completely unfolded, we firmly believe that immune phenotyping should be mandatory in all patients with suspected or proven peroxisome disorders.

In conclusion, although the findings described in this case report do not demonstrate a cause–effect connection between ZS and any form of PID, we think that the description of these clinical cases will increase the knowledge about the syndrome in object and will offer food for thought on the management of those patients. Moreover, it could represent an interesting starting point for further studies on peroxisome dysfunction and their causative role in pathogenesis of humoral and cellular immunodeficiency.

## Data Availability Statement

The datasets for this article are not publicly available due to concerns regarding participant/patient anonymity. Requests to access the datasets should be directed to the corresponding author.

## Ethics Statement

Ethical review and approval was not required for the study on human participants in accordance with the local legislation and institutional requirements. Written informed consent to participate in this study was provided by the participants' legal guardian/next of kin. Written informed consent was obtained from the minor(s)' legal guardian/next of kin for the publication of any potentially identifiable images or data included in this article.

## Author Contributions

SR: conceptualization. CF: writing—original draft preparation. All authors: writing—review and editing. SR, CF, and LM: data curation. CA: expert immunology field. EP, MD, and FP: expert metabolic diseases field. All authors contributed to manuscript revision, read, and approved the submitted version.

## Funding

This work was supported in the context of Newborn Screening for Primary Immunodeficiency project funded by FONDAZIONE CASSA DI RISPARMIO DI FIRENZE - MORIONDO_FCRF_2017.0786.

## Conflict of Interest

The authors declare that the research was conducted in the absence of any commercial or financial relationships that could be construed as a potential conflict of interest.

## Publisher's Note

All claims expressed in this article are solely those of the authors and do not necessarily represent those of their affiliated organizations, or those of the publisher, the editors and the reviewers. Any product that may be evaluated in this article, or claim that may be made by its manufacturer, is not guaranteed or endorsed by the publisher.
